# Sustained high trough factor IX activity levels with continued use of rIX‐FP in adult and paediatric patients with haemophilia B

**DOI:** 10.1111/hae.13735

**Published:** 2019-03-13

**Authors:** Joan C. Gill, John Roberts, Yanyan Li, Giancarlo Castaman

**Affiliations:** ^1^ Blood Research Institute, Blood Center of Wisconsin, Medical College of Wisconsin Children's Research Institute Milwaukee Wisconsin; ^2^ CSL Behring King of Prussia Pennsylvania; ^3^ Center for Bleeding Disorders and Coagulation Careggi University Hospital Florence Italy; ^4^ Department of Cell Therapy and Hematology, Hemophilia and Thrombosis Center San Bortolo Hospital Vicenza Italy

Bleeding severity in haemophilia usually correlates with the levels of circulating clotting factor.^1^ Patients with mild (factor levels 6%‐40% of normal activity) or moderate haemophilia (1%‐5%) bleed infrequently, predominantly due to trauma or after surgery, while individuals with severe disease (<1%) experience frequent spontaneous bleeding, particularly into joints and muscles, or after minor trauma.^1^ In patients with haemophilia B, prophylaxis with factor IX (FIX) aims to maintain appropriate trough levels to reduce the incidence of bleeds.^1^ In severe cases, lower trough levels are associated with an increase in total bleeding events and haemarthroses.^2^

rIX‐FP (albutrepenonacog alfa, IDELVION^®^, CSL Behring, King of Prussia, PA, US) is a fusion protein genetically linking recombinant human coagulation FIX with recombinant human albumin and has an extended half‐life compared with standard products, allowing a prolonged dosing interval.^3^ The safety and efficacy of rIX‐FP was demonstrated in adults/adolescents (7‐, 10‐ and 14‐day prophylaxis treatment regimens) and paediatrics (7‐day prophylaxis only) in two phase III trials of previously treated patients (PTPs) with severe haemophilia B (FIX activity [FIX:C] ≤2%).^3^, ^4^

The aim of this analysis was to evaluate mean steady‐state and observed trough FIX:C levels during prophylaxis with rIX‐FP in these two clinical trials and to assess the impact of treatment with rIX‐FP on patients with haemophilia B.

The detailed study designs of the adult/adolescent (NCT0101496274) and paediatric (NCT01662531) rIX‐FP phase 3 studies have been described previously.^3^, ^4^ Subjects were male PTPs with haemophilia B (FIX ≤2%) without inhibitors, and informed consent was acquired from all patients. Adult and adolescent patients (≥12 years) received prophylaxis with 35‐50 IU/kg rIX‐FP every 7 days or 50‐75 IU/kg every 10 or 14 days; paediatric patients (<12 years) received prophylaxis with 35‐50 IU/kg rIX‐FP every 7 days.^3^, ^4^ This analysis is limited to 7‐ and 14‐day regimens.

FIX:C was measured at a central laboratory using a validated one‐stage clotting method with Pathromtin SL (Siemens Healthcare Diagnostics, Marburg, Germany) as an activator agent, as previously described.^3^ Adult/adolescent trough FIX:C levels were measured every 4 weeks before each infusion over a maximum period of approximately 70 weeks; paediatric trough FIX:C levels were measured at 4, 12, 24 and 36 weeks.

Steady‐state trough FIX:C included only FIX trough measurements after three consecutive doses on the 7‐day regimen and two consecutive doses on the 14‐day regimen; if unscheduled doses of FIX product were administered, subsequent trough FIX measurements occurring within 21 and 28 days for the 7‐day and 14‐day regimens, respectively, were excluded. Thus, steady‐state trough FIX:C levels reflect the FIX:C values achieved after repeated dosing, where FIX:C levels have reached their maximum plasma concentration for a dosing regimen.

Observed trough reflects the measured FIX:C levels at a given time point and includes data from all patients with at least one measurement obtained at observed trough during prophylaxis treatment. Observed trough FIX:C measured during pharmacokinetic (PK), repeated PK or surgical periods, or during an unscheduled visit was not included; if an additional dose was given (eg to treat a bleed) between two prophylaxis doses, the observed FIX:C measurement was excluded.

Ninety adult/adolescent (n = 63) and paediatric (n = 27) patients with severe haemophilia B (≤2% FIX) were enrolled in the studies. Two paediatric patients did not participate in the PK analysis. The majority of patients had previously received prophylaxis (71.1%). Baseline demographic data have previously been published.^3^, ^4^

Adults/adolescents (n = 33) receiving 35‐50 IU/kg rIX‐FP prophylaxis every 7 days had a mean steady‐state trough FIX:C level of 20.90% (Table 1, Figure 1A), with a mean dose of 41.3 IU/kg. Adults/adolescents (n = 16) receiving 50‐75 IU/kg rIX‐FP prophylaxis every 14 days had a mean steady‐state trough FIX:C level of 12.76% (Table 1, Figure 1B), with a mean dose of 73.5 IU/kg. Including all dose levels, the mean FIX:C trough levels were 22.26% and 12.48% for 7‐day and 14‐day regimens, respectively. Nearly all (96.2%) steady‐state trough measurements were above 5% across all doses and dose intervals. The observed trough values were similar, with mean levels of 22.09% and 12.37% for 7‐day and 14‐day regimens, respectively. Adults/adolescents had a median (IQR) annualized bleeding rate (ABR) of 0.00 (0.00, 1.87) on weekly prophylaxis and 1.08 (0.00, 2.70) on the 14‐day dosing interval. The median annualized spontaneous bleeding rate (AsBR) on all dosing regimens was 0.00 (0.00 [0.00, 0.00] on a 7‐day regimen and 0.00 [0.00, 1.00] on a 14‐day regimen).

**Table 1:** Summary of steady‐state and observed trough FIX:C across rIX‐FP dosing regimens

	Adult and adolescent patients					Paediatric patients			
	7‐day regimen			14‐day regimen		7‐day regimen			
	25‐40 IU/kg	35‐50 IU/kg	All doses	50‐75 IU/kg	All doses	25‐40 IU/kg	35‐50 IU/kg	40‐55 IU/kg	All doses
	Steady‐state trough FIX, %								
N subjects (N measurements)	20 (89)	33 (156)	41 (230)	16 (83)	18 (106)	6 (13)	16 (27)	17 (30)	23 (55)
Mean (SD)	19.63 (5.81)	20.90 (6.39)	22.26 (7.08)	12.76 (6.80)	12.48 (6.36)	11.02 (4.09)	12.80 (4.17)	12.59 (4.27)	12.15 (4.15)
Median	20.70	21.75	22.60	12.40	12.40	11.50	13.10	12.60	12.90
Min, max	5.0, 33.4	5.0, 38.6	5.0, 50.4	3.1, 50.2	3.1, 50.2	3.2, 17.5	5.0, 24.1	5.0, 24.1	0.7, 24.1
	Observed trough FIX, %								
N subjects (N measurements)	20 (101)	37 (185)	45 (278)	16 (88)	18 (113)	8 (18)	19 (41)	19 (43)	25 (74)
Mean (SD)	19.39 (5.92)	20.49 (6.48)	22.09 (7.21)	12.71 (6.72)	12.37 (6.30)	10.39 (4.12)	12.40 (4.36)	12.57 (4.41)	12.11 (4.40)
Median	20.50	21.30	22.35	12.40	12.40	10.50	11.70	12.10	12.30
Min, max	5.0, 35.3	5.0, 38.6	5.0, 50.4	3.1, 50.2	3.1, 50.2	3.2, 17.5	5.0, 24.1	5.0, 24.1	0.7, 24.1

FIX, factor IX; min, minimum; max, maximum; N, number; SD, standard deviation.

**Figure 1:**
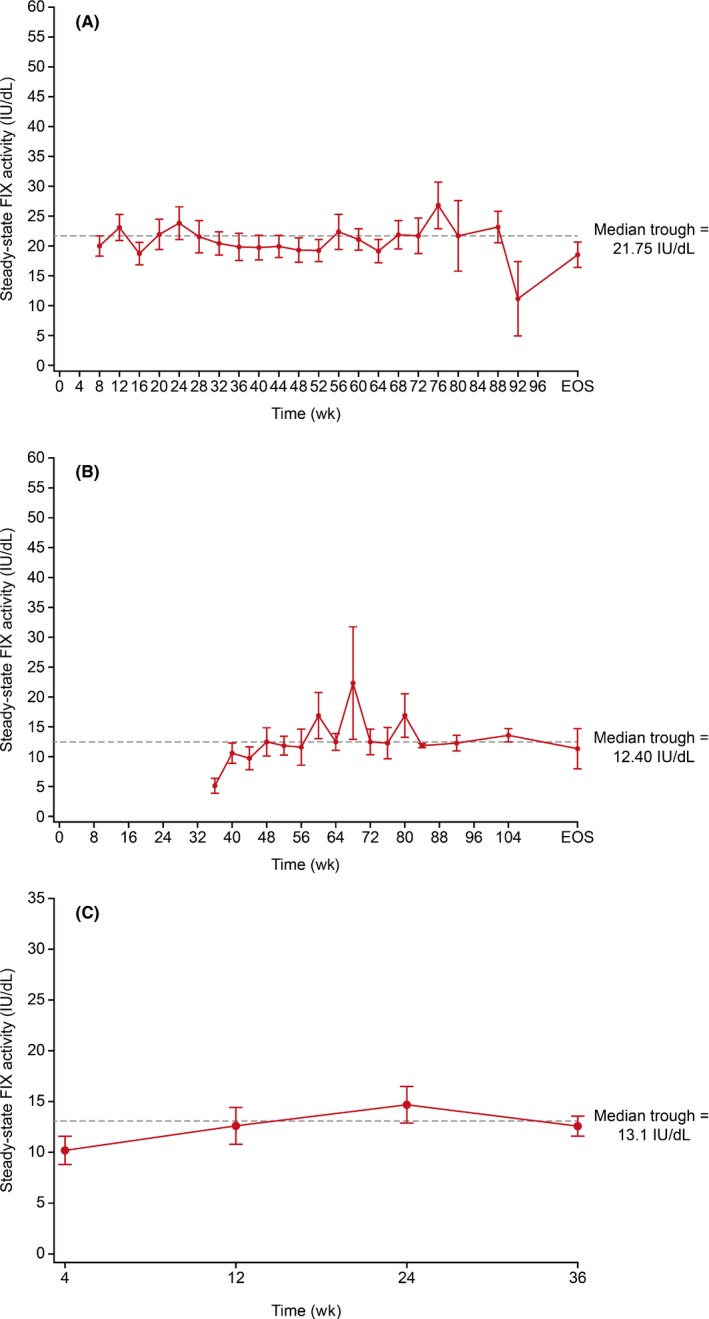
Mean (±SE) steady‐state FIX:C trough levels. In patients aged ≥12 years, 35‐50 IU/kg rIX‐FP every 7 days (A) and 50‐75 IU/kg rIX‐FP every 14 days (B). In patients <12 years, 35‐50 IU/kg rIX‐FP every 7 days (C). (A) There were at least three observations at each timepoint, except at 92 hours where data from only two observations were available. (B) There were at least two observations at each timepoint

Sixteen paediatric patients received 35‐50 IU/kg rIX‐FP prophylaxis every 7 days with a mean dose of 43.8 IU/kg and a mean steady‐state FIX:C trough level of 12.80% (Table 1, Figure 1C). The mean observed trough was similar at 12.40%. Across all doses, 97.9% of steady‐state trough measurements were above 5%. The median (IQR) ABR for all patients in the paediatric study was 3.12 (0.91, 5.91); median AsBR was 0.00 (0.00, 0.91).

rIX‐FP delivers consistently high observed trough FIX:C levels with 7‐ and 14‐day prophylaxis dosing regimens. These findings are consistent with the low median ABRs observed^3^, ^4^ and suggest that by maintaining high trough FIX:C levels, rIX‐FP effectively converts a patient with severe haemophilia B to a mild bleeding phenotype. The 7‐ and 14‐day prophylaxis regimens, albeit at different dose ranges, result in FIX trough levels >10%, suggesting that in adult patients therapy can be individualized to suit a patient's lifestyle and requirements, depending on the trough level required.

One limitation of studying FIX trough levels is that FIX enters the extravascular space, and only plasma FIX:C levels can currently be measured. The extravascular space may act as a store for FIX; therefore, trough levels may not correlate directly with the amount of FIX present in tissues nor the ability of FIX to prevent bleeds.^5^ In biodistribution studies, rIX‐FP was detected in extravascular mineralized bone regions 120‐240 hours postdose, whereas conventional recombinant FIX (rFIX) was not visible after 24 hours.^6^ The excellent haemostatic efficacy of rIX‐FP at extended dosing intervals may be due to the FIX stored in the extravascular space.^5^ Further study of extravascular FIX and its association with FIX trough levels and bleeding rates is required.

Trough levels for other extended half‐life products have been evaluated; however, studies between the products cannot be directly compared, due to differences in the study populations. An rFIX Fc fusion protein (rFIXFc; ALPROLIX^®^, eftrenonacog alfa; Biogen Idec, Inc, Cambridge, MA) produces a low ABR with prophylaxis every 1‐2 weeks.^7^ Dosing in clinical trials was designed to target trough levels of 1%‐3%,^7^ and lower bleeding rates were seen with increasing trough levels^2^; however, steady‐state trough FIX:C levels have not been reported. rIX‐FP delivers steady‐state trough FIX:C levels >10% with similar dosing regimens, which may provide additional protection from bleeds. Indeed, clinical studies have shown that the AsBR in adult/adolescent patients was 0 with rIX‐FP, but with rFIXFc was 1.0 and 0.9 with weekly and individualized prophylaxis, respectively.^3^, ^7^ PK data for another extended half‐life product, glycopegylated rFIX (N9‐GP, Refixia^®^, nonacog beta pegol; Novo Nordisk, Bagsvaerd, Denmark) indicate that weekly treatment with 40 IU/kg N9‐GP provides predicted mean trough FIX:C levels of 31% in adult/adolescent patients and 13% for paediatric patients.^8^ Imaging studies in haemophilia B mice indicate that N9‐GP may remain in the plasma and might not enter the extravascular space.^9^ More studies are required to confirm whether this also applies to human tissues. However, this difference in distribution may explain the higher FIX:C levels but comparable efficacy observed with N9‐GP; both N9‐GP and rIX‐FP have AsBRs of 0 in adult and paediatric patients.^3^, ^4^, ^10^ Of patients treated with rIX‐FP, >95% maintained a mild haemophilia phenotype (FIX:C > 5%) at all time points and on all regimens. Patients may find that an extended dosing regimen would still provide protection from bleeds but be easier to maintain.

rIX‐FP provides consistently high steady‐state trough FIX:C levels with 7‐ and 14‐day prophylaxis regimens contributing to median AsBRs of 0. These findings demonstrate the successful transition of the majority of patients with severe haemophilia to a mild haemophilia phenotype. With rIX‐FP, patients benefit from flexible dosing with very high protection against bleeds.

## DISCLOSURES

This work was supported by CSL Behring. GC and JCG have served on advisory boards for CSL Behring. JR and YL are employees of CSL Behring. GC has served on advisory boards or as a meeting speaker for Kedrion, Pfizer, Shire, Bayer, Novo Nordisk, Roche, Uniqure and Sobi.

## AUTHOR CONTRIBUTION

JR and YL contributed to the investigation and formal analysis of the data; GC and JCG contributed to the investigation and resourcing of the study; all authors were involved in preparing the original draft of the manuscript and revising it critically and gave final approval of the version to be published.

## Data Availability

Individual participant data will not be shared as patient anonymity cannot be ensured.
